# Implementation and knowledge translation of health promotion programs at scale: “a perspective article”

**DOI:** 10.3389/fpubh.2026.1853616

**Published:** 2026-08-28

**Authors:** Hatem H. Alsaqqa

**Affiliations:** 1 Faculty of Public Health and Deanship of Scientific Research, Al-Quds University, Jerusalem, Palestine; 2 Palestinian Ministry of Health, Gaza, Palestine

**Keywords:** health promotion, implementation, knowledge translation, ontology, program, scale

## Abstract

Health promotion decision-makers must decide which evidence-based health programs will advance the health of a target population regarding a specific health concern. A key component of evidence-based implementation is the complexity of health programs, which means that numerous factors affect their outcomes. However, the level to which the results of a successful health program assessed in a primary context can be attained in a target context is known as transferability. Even when there is evidence of their value or efficacy, the strategies for large-scale public health program implementation received much less attention. Thus, understanding how successful interventions are expanded from research settings into more general policy and practice is made easier with the help of theoretical frameworks. Therefore, the drivers and challenges that can affect the translation and adoption of research evidence in health policy and practice are highlighted in this perspective paper. Effective legislation and practice depend on pertinent and useful evidence, which decision-makers integrate into plans and programs to increase health system coverage and enhance health outcomes. Although numerous health promotion interventions have demonstrated effectiveness, the translation of research evidence into large-scale implementation remains fragmented. This perspective proposes ontology-based knowledge as an innovative approach to strengthen evidence synthesis, knowledge translation, reusable implementation knowledge. Ontologies offer unifying frameworks that can promote more efficient knowledge accumulation by supporting the synthesis of researchers' empirical work in a knowledge base, ultimately facilitating the translation of this evidence into actionable health policies and practices.

## Introduction

Nutrition, exercise, sexual health, and interventional programs related to alcohol, tobacco, and drug use can all improve health and decrease morbidity and mortality (1). In order to improve community health and prevent disease, health promotion and prevention research is essential. The application of the best available scientific evidence to guide public health policy and practice to enhance community health is, in fact, the cornerstone of the evidence-based health paradigm. A variety of possible effects that health research may have on health promotion policy or practice are listed in research impact frameworks, such as government adoption, or use to guide policy or judicial decisions (2).

Increased use of evidence-based health promotion is anticipated to have several advantages, such as better information about best practices for decision-makers, a greater chance of successful policy and program implementation, and more effective use of resources (3, 4). Moreover, international, national, and local decision-makers, population health researchers, practitioners, and stakeholders will be impacted by a program are user groups for evidence-based health promotion.

Even though estimates of how research affects public health policy and practice vary greatly, taken as a whole, they seem to be higher than what is typically reported in other medical specialties, indicating that 14% of health innovations have an impact on policy or practice, with an average time of 17 years (5). This could be because different studies use different methodological ways to gauge research translation.

Furthermore, policies, and resource distribution strategies (6), community health programs (7), and diagnostic, therapeutic, cognitive, and other health care interventions and services (8) are just a few examples of the various levels at which health promotion interventions can be put into practice.

A significant barrier to population-wide health improvement is the inability of successful public health programs to impact public health practice as well as the delay between the creation and use of evidence, which prevents or postpones community access to efficient services (9). However, the process of expanding health programs that have been demonstrated to be effective on a limited scale and/or under controlled conditions into broader policy or practice under real-world situations is known as “scaling up” (10). The idea of scaling up entails a deliberate plan to extend an intervention's reach to new contexts or target populations, along with a methodical approach to accomplish this goal (11).

In order to describe and comprehend how successful interventions are expanded from small trials into larger policy and practice, theoretical frameworks and approaches can be helpful. They can also serve as valuable support tools for practitioners and policy makers as they work to expand public health programs. In the fields of public health, these evidence-to-practice frameworks are becoming more and more common (10).

The term “translation” is synonymous with “transfer,” which means “to carry across” and refers to the process of transferring an intervention, such as how it is put into effect (12). Over the past 20 years, the area of implementation science has grown rapidly as more evidence has been produced to support methods for successfully, sustainably, and widely integrating research findings into health practice and policy (13). Simultaneously, there is a rising awareness of the necessity of building capacity among health professionals and within healthcare settings to support evidence-based knowledge translation (KT) processes and facilitate the timely, consistent, and sustained application of research evidence in health practice (14, 15).

Furthermore, in order to design and implement KT interventions, cooperative partnerships between knowledge producers (such universities), knowledge brokers, and non-researchers are essential (16). By guaranteeing that information is co-created with people who will finally use it, improving research applicability and implementation, and promoting sustainable outcomes, co-production of knowledge in KT closes the gap between research and practice (17).

In order to ensure direct application to strengthen health policies and practices, collaborative approaches like Integrated KT stress the co-production of evidence by researchers, policymakers, and practitioners along the research process (18).

Education programs in the field of implementation and dissemination science have emerged as a result of this acknowledgment; many of these programs have been spearheaded by universities and target academic researchers (19). It also incorporates interactive learning methods such as policy games, knowledge brokers, communities of practices and learning, partnership and network strategies, and sharing knowledge for translation (20). The sustainability of healthcare delivery systems depends on increasing the capacity and competence of healthcare services and health professionals to accept, modify, and apply research results (14).

Nonetheless, large-scale implementation in health promotion refers to the systematic expansion of evidence-based interventions to reach broader populations while maintaining effectiveness, quality, and sustainability across diverse contexts. It involves coordinated efforts to integrate health promotion strategies into existing health systems, policies, and community structures, ensuring that interventions can be delivered consistently at regional, national, or international levels.

Successful large-scale implementation depends on factors such as organizational capacity, stakeholder engagement, leadership, adequate resources, and continuous monitoring and adaptation to local contexts (21). Implementation science frameworks further emphasize that scaling up requires balancing fidelity to the original intervention with necessary contextual adaptations to maximize population health impact (22).

This perspective primarily considers implementation of health promotion programs in high-income countries, where substantial investments in health promotion coexist with persistent implementation and sustainability challenges. Although examples are drawn primarily from high-income countries, the conceptual principles are intended to be transferable to other health system contexts.

## Between policymakers and researchers

One important reason for this persistent disconnect is that implementation evidence is rarely organized in a form that policymakers can readily compare across programs (23). Although effectiveness evidence continues to accumulate, implementation information—including contextual influences, implementation strategies, fidelity, adaptation, and sustainability—is reported inconsistently. Implementation ontologies combined with evidence-informed rating systems offer a common language that can bridge researchers, practitioners, and policymakers.

This gap is also exacerbated by more pragmatic problems, such as a lack of networking or collaborative relationships between researchers, policymakers, and practitioners, time constraints, and knowledge of pertinent research (24). If the policy-making process is to advantage from the use of timely and practice-relevant research, certain challenges may arise and must be overcome.

In order to produce significant results on policy, services, systems, and decision-making, research evidence should be more widely acknowledged as a catalyst. Publications in the brief would further highlight the crucial significance of research evidence in creating workable solutions, even though there is already a strong foundation of information regarding obstacles to evidence-based policy and practice. It will accomplish this by directing the creation, execution, and evaluation of policies that address recognized issues and gauge their practical effects.

Through knowledge co-production and assessment, academics can better identify practice and policy priorities as a chance to collaborate with external stakeholders and support changes in practice, policy, or systems. Different priorities, languages, and timetables among health promotion actors, as well as conflicting influences from other sectors, frequently overshadow the use of research evidence in decision-making. Since research findings rarely communicate for themselves, it is crucial for academics and researchers to evaluate the data and effectively communicate the produced knowledge to advocate for action.

Nonetheless, there is often little interaction between the policy-making and research groups as they work in isolated networks (25). The academic research system and the bureaucratic policy-making system, in particular, can function independently without appropriate exchange of ideas, knowledge, or viewpoints because institutional and political forces are so dissimilar. For better societal and community outcomes, this systemic gap must be closed.

## The scope of translation process

It is important to view the process of translating research into practice and policy as a process rather than an end result. At the conclusion of the research process, publishing and the incorporation of research findings into practice or policy are typically used to define research success and impact. Research to policy translation is often thought to happen only after the research is concluded. It is becoming increasingly clear that translation is an ongoing, iterative process that might start at the very beginning of a concept (26). Collaborating with researchers, policymakers, and practitioners to examine the concept from multiple perspectives fosters fresh ideas, which in turn leads to the formulation of policy-relevant research topics that address contemporary policy issues.

However, research translation is iterative and cyclical, continuing throughout the research and policy-making process to produce evidence that can be utilized for addressing societal needs and to be responsive to changing political climates, as opposed to considering translation and impact at the end of the research process.

Engaging stakeholders early and continuously fosters trust and creates new opportunities.

The element of trust built in formal and informal partnerships is a crucial prerequisite for achieving efficient information exchange, where the best available evidence informs policies. Despite thrilling pressure, strong ties enable policymakers to take study findings into interpretation when making choices. Ultimately, helping policy actors ask the appropriate questions is essential to successful translation (27).

Moreover, the research community can adopt a more adaptable and iterative strategy to surge the influence of research on policy-making and implementation. Researchers can incorporate iterative revisions and modify their questions and methodologies based on findings and stakeholder situations, instead of expecting that their work would follow a methodically linear approach from hypothesis formulation and testing to conclusion (26).

By accepting that research is a non-linear process, they might see obstacles and unforeseen turns as chances to improve techniques and inquiries. Repositioning research findings as a crucial resource to support and enlighten policy-making and implementation is made possible by setting reasonable expectations and appreciating this ongoing improvement. This strategy promotes a culture that prioritizes quality and relevance in addition to publication volume, resulting in more significant contributions to society and knowledge as well as a decrease in research waste. In addition to helping academics dispel these widespread misconceptions, the new brief and its potential publications will highlight effective research translation strategies (26).

Despite considerable advances in implementation science and knowledge translation, these fields remain insufficiently connected in ways that support routine decision-making for health promotion at scale. Existing implementation frameworks explain determinants of implementation, while knowledge translation frameworks facilitate evidence uptake; however, neither provides a standardized infrastructure for systematically identifying, comparing, and communicating the implementation characteristics of health promotion programs (22). Implementation rating systems supported by standardized implementation ontologies have the potential to provide this missing infrastructure by enabling consistent classification of implementation evidence, improving evidence synthesis, and supporting scalable policy decisions.

## The required intersection for health promotion policy-making

The intersection of policy-making, research, and health policy-making is the process by which governments allocate funds and resources to implement public policy outcomes in communities, thereby translating their political vision into concrete programs, services, and actions. Making policies is often presented as a systematic, cyclical process that begins with the identification of and proceeds with an issue to the assessment of the implemented solution, which is usually in the form of a program, service, or structural modification.

Therefore, researchers must actively engage in the policy-making process for health promotion programs to be successful, and policymakers should actively strive to include researchers at every level of the policy cycle. Essentially, any government, at any stage, is measured by its policy decisions and implications, which puts a high premium on enhancing decision-making as a mechanism to produce the desired results (28). Compared to traditional policy formation processes, which may be limited by time and political factors and may not have a solid evidence base, an evidence-informed approach to policymaking is more successful and affordable (29).

Although the World Health Organization (WHO) Guidelines on Physical Activity and Sedentary Behavior provide globally applicable recommendations for physical activity promotion (30), their translation into policy and practice remains fragmented across high-income countries (22). Countries differ substantially in how the guidelines are implemented through national policies, healthcare systems, community programs, workplace initiatives, and funding mechanisms, resulting in considerable variation in implementation fidelity, population reach, and long-term sustainability (22, 31).

Evidence on effective implementation is often context-specific, limiting opportunities to identify transferable implementation strategies and the causal mechanisms that support successful scale-up and long-term sustainment across diverse high-income settings (32).

Subsequently, policymakers can direct decision-making and guarantee the sustainability and impact of their health promotion programs by using evidence-based strategies throughout the policy cycle. However, as the field of health promotion has grown to address more complex societal issues like poverty and social injustice, the digital and commercial determinants of health, and the social determinants of health equity, the challenges of converting evidence into effective and significant policy and practice are also growing (26).

## Effectiveness rating system

In a conventional evidence-based approach, local users are encouraged to apply the most successful programs once the available evidence for their efficacy is evaluated centrally. This method operationalizes quality as an intervention's demonstrated efficacy and views interventions as transportable packages. Health professionals, policymakers, and others can benefit from intervention development, research, and appraisal work that has been done elsewhere and improve the efficiency and legitimacy of health promotion by having experts rate the effectiveness of programs centrally (33).

Current effectiveness rating systems primarily evaluate intervention outcomes while providing limited information regarding implementation quality, contextual adaptation, implementation strategies, and long-term sustainability (34). Consequently, interventions with similar effectiveness ratings may differ substantially in their likelihood of successful implementation at scale (32). Integrating implementation ontologies into effectiveness rating systems would allow decision-makers to evaluate not only whether interventions work, but also how, why, for whom, and under what contextual conditions they can be successfully implemented and sustained.

Therefore, a rating system may make learning in the health promotion system easier by providing a space for standards to be articulated and for knowledge to be shared and integrated (35). Efforts to apply an evidence-based approach to lifestyle-related health promotion have encountered numerous difficulties, despite the fact that an evidence-based approach to health promotion appears auspicious and may deliver significant advantages (33).

The problem of evaluating impacts and defining their relevance is further complicated by the fact that linked changes in health may take a long time to occur in addition to interdependence and multi-causality (36). However, the promise of an evidence-based approach and the difficulties faced have spurred the search for more effective ways to use the central rating of efficacy in health promotion (37). The Effectiveness Rating System (ERS), often called the Recognition System, is an intriguing effort to adapt the concepts of the evidence-based movement to health promotion (38).

## Knowledge translation frameworks of health promotion

A variety of KT frameworks, such as the push-pull capacity model (39) and the framework for KT (40), have been created to assist research translation. It may be possible to determine where research impact is more expected and where extra tactics might be needed to enhance the translation of evidence for public health and community support by knowing the features of impact-related research.

Existing implementation frameworks have substantially improved understanding of implementation determinants and processes. Nevertheless, they are primarily designed to explain implementation rather than organize implementation knowledge into forms that support evidence synthesis and policy decision-making. However, valuable implementation knowledge remains dispersed across studies and difficult to compare (41).

As well, tools like theories, models, or frameworks (TMFs) can be used to summarize and disseminate information and evidence. The KT process is explained using TMFs in terms of system-level modifications, organizational preparedness, and individual practices (42). For KT interventions in health systems, the local context, culture, and environment are crucial. If involved stakeholders are asked for their opinions, KT strategies are more likely to be applicable and helpful (43). Co-produced evidence is frequently more acceptable, feasible, and likely to result in long-lasting change (44). Furthermore, the cultural context and demands of information users can influence the need for research evidence.

Evidence, strategies, platforms, tools, stakeholders, and mechanisms are all part of KT interventions and activities. KT techniques are methods intended to address research-practice gaps (i.e., what we know vs. what we do) and reassure the adoption of evidence-based interventions in healthcare practices and policy (45). KT strategies can target different levels (individual, team, system), be single or multifunctional, and concentrate on the acceptance, implementation, and/or sustainability of an evidence-based program (23).

Moreover, TMFs for integrating evidence into health practice and policy were found in a recent scoping review (46). It has also been suggested that broad models of KT frameworks be enhanced with perceptions from political science and policy theory, given the intricate political character of health policies (47). As a consequence, Langer and colleagues (48) carried out a systematic scoping review of reviews pertaining to the application of research evidence in decision-making more generally, encompassing decision-makers from various levels and contexts, such as those with backgrounds in social science, political science, education, or health (48). Using “push,” “pull,” and/or “exchange” tactics, they recognized six fundamental mechanisms through which they hypothesized KT interventions intended to improve evidence-informed decision-making work, either alone or in combination (49).

KT frameworks successfully describe how evidence moves from research into practice; however, they rarely specify how heterogeneous implementation evidence should be systematically organized, classified, or compared across interventions (22). As a result, implementation knowledge often remains disorganized despite extensive reporting. Implementation ontologies can address this limitation by structuring implementation evidence using standardized concepts that facilitate evidence synthesis, interoperability, and informed policy decisions (50, 51).

For the purpose of supporting the ultimate outcome of “research use,” Langer and colleagues (48) described how the six underlying mechanisms affect one or more elements of behavior change (i.e., capability, opportunity, or motivation). The SPIRIT framework postulates that when there is adequate capacity, a number of behaviors that simplify conceptual, instrumental, tactical, and/or imposed research use take place in the form of “research engagement actions,” such as accessing, evaluating, commissioning research, or cooperating with researchers (49).

However, programs were put into place at four structural impact levels (52). Research translation in health practice translation capacity-building programs. Leaders of KT capacity-building programs can maximize opportunities for continued funding by generating high-quality evaluations that show practice impact and program alignment with larger health policy agendas (e.g., promoting equity and quality in healthcare, and reducing inefficiencies) (53).

Furthermore, although they mostly concentrate on implementation science, an increasing number of frameworks for scaling up health innovations in the field of health promotion have been published (54). Different viewpoints for comprehending the mechanics underlying the scaling-up process can be obtained from implementation science and complexity science. Nonetheless, the author stresses the significance of a social science viewpoint for scaling up, as it may assist researchers to tap into the organizational and societal influences on scale-up.

In an effort to identify and comprehend the essential steps required for scaling up a health promotion program, framework creation was influenced by theories related to scale-up, such as complex adaptive systems, and diffusion of innovation (55). Nevertheless, frameworks or models should be able to guide decisions and judgments that arise during scaling-up processes (56). The ExpandNet conceptual framework (57), which aids in the strategic planning and management of the scale-up process, is one of the key resources published by ExpandNet, a network of public health professionals that was created in partnership with the WHO. Subsequent ExpandNet documents include scale-up planning (58), stages for creating a scale-up strategy, and helpful advice for scaling up health innovations (59).

## Collective impact on behaviors

Numerous academic disciplines, such as human development, sociology, and public health, employ socioecological models to comprehend the dynamic interrelationships among diverse individual and societal impacts on people's behaviors. The context, surroundings, or setting in which behaviors take place is frequently referred to as the collective level (60). Although they eventually become internalized, these influences are usually thought of as external to people (61). Strong circumstances put pressure on people to act in a particular way, according to situationism, which results in similar behaviors among people as unique thoughts, feelings, and behavior patterns are repressed (62).

However, in health promotion, socio-ecological models highlight how people are a part of various social systems and recognize how environments and people interact to affect health outcomes. Therefore, in order to comprehend and impact health behaviors, social-ecological models seek to concurrently concentrate on various aspects and their interconnections in various ecological systems (individual, interpersonal, and environmental) (63). This paradigm confirms that the environment and the individual interact to shape behavior, and that various levels—individual, social, physical, and political—are actively involved.

Supporting improved health through behavior modification and maintenance is a major objective of behavioral health. Behavior modification is a challenging scientific issue because human behavior is highly dynamic, multifactorial in origin, and influenced by interactions between individuals and contextual circumstances (64). Substantial methods for efficiently forming and curating scientific knowledge are needed to match this level of complexity in order to facilitate the aggregation and comparison of findings from various research (65, 66).

Currently, the lack of common words and labels hinders the effective acquisition of knowledge. Target behaviors like eating “healthily” and exercising, for instance, are measured in a variety of ways that may or may not match to the same fundamental phenomena (e.g., self-reported physical activity is often only weakly correlated with objective physical activity measurements). The behavioral literature frequently has this issue of terminology and labels being mixed together (67).

Nonetheless, different constructs may have the same label (e.g., objective and self-report physical activity are both labeled as physical activity, but they may represent different constructs). However, the same construct may be referred to by different names (e.g., self-efficacy vs. perceived behavioral control vs. locus of control). It is challenging to aggregate knowledge across behavioral science due to the lack of common terms and definitions for programs, mechanisms of action, outcome measures, and target context (67). Thus, the author believes that such updated advanced methods as ontology are essential to suitably study the complexity of human behavior.

## The potential of ontologies

Recent developments in the fields of behavioral, machine, and information sciences offer a significant shift in the opportunities for quickly, effectively, and efficiently answering complicated issues. Information science advancements include the automation of certain procedures, such as the assessment of trial bias risk. Techniques for making synthesized evidence available are developing, but they rely on the ability to come up with effective search keywords for the gathered information.

However, computing systems have made it possible to identify published reports of interventions more effectively (68), which has decreased the workload for humans and accelerated the synthesis of evidence. They also have the potential to more successfully integrate the evidence (69) and, in the long run, generate new perceptions by spotting patterns and connections that are missed by existing techniques.

A review of automated evidence extraction techniques, pointed out the approaches' shortcomings and emphasized the significance of having a solid framework or “ontology” for organizing the potentially collected data (70). An ontology is a methodical framework for arranging information about (1) entities (constructs) and (2) their interrelationships using a regulated vocabulary for labels and definitions. They make it easier to organize, reuse, integrate, and analyze this information by codifying it in a computer-readable way (67). Additionally, because computers can examine patterns in the evidence more quickly and without human presumptions, the systematic organization of knowledge by ontologies may result in the creation of extra knowledge that is there, but unrecognized in the evidence base.

Moreover, a strategy for better coordinated evidence extraction and synthesis is being made possible by developments in behavioral science that provide common vocabulary and conceptual frameworks for behavior change programs. Health promotion researchers will produce more systematic reviews if implementation science still relies on human integration of evidence using disparate terminologies. Thus, this approach is questionable to provide the theoretical understanding of behavior change that implementation researchers, practitioners, and the research, policy, and communities as a whole expect, particularly because it does not adequately address the complexities of human behavior and the contextual factors that influence it.

Nonetheless, the goal of the Wellcome Trust-funded Human Behavior Change Project, which runs from 2016 to 2020, is to advance scientific advancements in order to significantly improve our capacity to synthesize evidence at scale, speed, and granularity (50). In order to produce new insights about behavior change and increase the prediction of program effectiveness, it employs artificial intelligence and machine learning to create and assess a “knowledge system” that continuously scans the global literature on behavior change programs, automatically extracts important information, synthesizes, and interprets results (50).

## Conclusion

Health promotion programs will need to be expanded for wider adoption in order to improve population-wide health. Policymakers and senior decision-makers can obtain crucial information to support the broad adoption and sustaining of health promotion programs by taking into account aspects related to “scalability” during the design phase and throughout the intervention research continuum, from efficacy studies to dissemination studies.

As effective methods for quickly putting research into practice to enhance healthcare delivery systems and eventually health outcomes, are of growing importance to researchers, leaders, health policymakers, and health-research collaboratives. Researchers should adequately describe their work in terms of the population, program, environmental circumstances, procedures, and so on in order to assist transferability assessment.

Furthermore, taking into account appropriate variables for reporting may be aided by transferability criteria. Health promotion decision-makers require transferability information that is methodical and practically applicable. Thus, more studies are required to create a more effective tool for the models, assess this tool with various target audiences, and examine its practical utility.

Nonetheless, the author demonstrated that ontologies have comparable potential in behavioral science and pointed out that they have been utilized to speed scientific advancement in other fields. In light of findings, the author displays that the health behavioral change community uses and creates ontologies for behavior change therapies, utilizing knowledge from ontologies created in other pertinent scientific fields. The quality of existing research and, eventually, its usefulness for policymakers would improve if these concerns were taken into consideration when designing and reporting interventional program studies.

Future implementation research should move beyond documenting implementation barriers toward developing interoperable knowledge structures capable of supporting cumulative learning across programs. Researchers, policymakers, and funding agencies should invest in rating system and ontology-driven implementation science to improve evidence synthesis, implementation planning, and large-scale knowledge translation.

## Data Availability

The original contributions presented in the study are included in the article/supplementary material, further inquiries can be directed to the corresponding author.
